# A corticostriatal circuit mediates the switching of defensive responses to an approaching threat

**DOI:** 10.1038/s41398-026-04105-3

**Published:** 2026-05-20

**Authors:** Junye Ge, Pengfei Ren, Yaning Zhang, Baijun Chen, Yiwen Deng, Jinwei Xu, Ying Zang, Qian Xue, Shengxi Wu, Chuchu Qi, Wenting Wang

**Affiliations:** 1 Guangdong Provincial Key Laboratory of Brain Function and Disease, Department of Physiology, School of Medicine, Shenzhen campus of Sun Yat-Sen University, 66 Gongchang road, Shenzhen, Guangdong China; 2 Department of Neurobiology, School of Basic Medicine, Fourth Military Medical University, Xi’an, China

**Keywords:** Neuroscience, Diseases

## Abstract

Defensive responses are evolutionarily conserved adaptive behaviors that species exhibit in response to threats to protect themselves from harm or death. The selection of context-appropriate defensive strategies, particularly the transition between freezing and flight behaviors, constitutes a critical determinant of species survival. We first established a two pure tone serial-compound stimulus (TTSCS) paradigm to evaluate the dynamic transition of defensive behaviors evoked by conditioned stimuli. This paradigm minimizes potential confounding effects on defensive behavior caused by differences in tone. Using this approach, we found that as the threat stimulus approached more closely, mice exhibited increased escape behaviors characterized by shorter latencies and larger magnitudes of response. These behavioral changes were associated with the dynamic responses of dopamine receptor 1 expressing MSNs (D1 MSNs) and dopamine receptor 2 expressing MSNs (D2 MSNs) in the dorsomedial striatum (DMS). Following functional validation through manipulation of the PFC (prefrontal cortex)–DMS circuit, we found that activating the PFC-DMS^D1^ pathway significantly shortened the response latency of mice to heightened threat stimuli, enabling faster defensive reactions. However, activation of D2 MSNs subtypes of PFC-DMS pathway resulted in no significant alterations in the defensive behaviors. In conclusion, D1and D2 MSNs in the DMS likely act in an antagonistic manner to regulate defensive behaviors, and furthermore, the PFC projection specifically to the direct pathway within the DMS mediates the shift in defensive responses to an approaching threat.

## Introduction

Defensive behaviors, shaped by natural selection, are automatically activated in humans and other animals to mitigate harm or mortality in dangerous situations [1]. When confronted with a threat, organisms display a flexible transition between different defensive responses [2]. Active responses, such as flight, cost more energy and carry greater risks. On the contrary, freezing is a common conservative defensive response, while it means forgoing opportunities to forage or seek potential mates. Therefore, the ability to promptly and accurately switch between freezing and flight in response to approaching threats is critical for survival [3].

The selection of defensive response is governed by a dynamic process known as risk assessment (RA). RA involves the evaluation of multiple factors, including the threat proximity on a spatiotemporal scale, which determines the transition between defensive behaviors [4, 5]. For example, freezing is typically the initial response to a realized threat (post-encounter mode), whereas flight emerges as the threat becomes imminent (circa-strike defensive mode). This pattern has been consistently observed in both humans and rodents exposed to naturalistic predator threats [2, 4, 6]. However, naturalistic predator threats make it difficult to disentangle the roles of stimulus type and threat proximity in driving defensive behaviors. To address this limitation, Fadok and colleagues developed a modified auditory Pavlovian fear conditioning procedure using a serial-compound stimulus (SCS) [7]. The SCS consists of a pure tone followed immediately by white noise, each eliciting distinct conditioned responses: freezing to the tone and flight to the white noise. After repeated trials, mice exhibit clear behavioral differentiation, freezing in response to the tone but fleeing upon hearing the white noise. However, whether these responses are driven by the auditory stimulus itself or by the perceived threat proximity encoded within the SCS remains debated [8, 9].

The transition between freezing and flight during the SCS involves stimulus–response learning, risk assessment, and action initiation. The dorsal striatum (DS) particularly its medial subdivision (DMS), has emerged as a key candidate for mediating these processes due to its well-established roles in stimulus–response learning, goal-directed behavior, and action initiation [10–16]. Emerging evidence further highlights the distinct roles of dopamine receptor 1 expressing MSNs (D1 MSNs) and dopamine receptor 2 expressing MSNs (D2 MSNs) in the DMS, with the DMS being critical for evaluating options and making decisions prior to outcome delivery [17–22]. Furthermore, Numerous studies have reported that the DMS receives strong projections from the prefrontal cortex (PFC) [23, 24]. The PFC serving as an association cortex involved in cognitive decision-making, assesses the risk posed by potential or actual threat stimuli in the environment [25–27]. It can dynamically encode threat features and initiate active avoidance [28], balance freezing and avoidance via inhibition of the central amygdala [28], and facilitate flexible behavioral switching through regulation of the uncinate fasciculus [29]. We therefore propose that during an approaching threat, the PFC may adaptively regulate defensive behavior by modulating the balance between the direct and indirect pathways in the DMS.

In this study, we developed a two pure tone serial-compound stimulus (TTSCS) paradigm building upon the classic SCS model, to evaluate the dynamic transition of defensive behaviors evoked by conditioned stimuli. It minimizes the unconditioned response to a sudden auditory stimulus change. We then examined changes in the activity of D1 and D2 MSNs in the DMS during exposure to progressively intensifying threat stimuli. By performing synchronous correlation analysis between behavioral responses and neuronal calcium dynamics, we sought to determine how D1 and D2 MSNs contribute to threat processing and the organization of defensive behaviors. Furthermore, using optogenetic manipulation combined with transsynaptic anterograde tracing, we identified the causal involvement of postsynaptic D1 and D2 MSNs within the PFC–DMS circuit in mediating defensive behavior. Our findings elucidate the distinct contributions of the direct and indirect pathways in the DMS to defensive behavior and underscore the critical role of PFC inputs to the direct pathway in enabling flexible switching between defensive responses.

## Methods

### Animals

All experimental procedures were approved by the Institutional Animal Care and Use Committee of the Fourth Military Medical University (Approval No. IACUC-20230320) and conformed to the Guide for the Care and Use of Laboratory Animals published by the National Institutes of Health. All mice were maintained under a 12-h light/dark cycle at 22–25 °C with food and water under environmentally controlled conditions. C57BL/6J mice were purchased from the Experimental Animal Center of the Fourth Military Medical University. The Drd1-Cre (#037156-JAX) and Adora2a-Cre (#031168-UCD) were obtained from the Jackson Laboratory. All virus injections were administered to mice aged 2 months old, and all behavioral tests were carried out during the light phase. The experimenters were blinded to the genotype and experimental conditions. All the mice employed in the behavioral tests were male.

### Virus injection and stereotaxic surgery

Mice were anesthetized with isoflurane (4% for induction and 1.5% for maintenance), and their heads were fixed in a stereotaxic injection frame (RWD Life Science Inc., China). Injections into the brain were performed using a microinjection needle with a 10 μL microsyringe (Shanghai Gaoge Industry and Trade Co., LTD., China) to deliver the 250 nl of virus at a rate of 30 nL/min using a micro syringe pump (KD Scientific Inc., USA). Following injection, the needle was held at the site for another 15 min to allow for diffusion. The coordinates were defined as dorsal-ventral (DV) from the skull surface, anterior-posterior (AP) from bregma, and medio-lateral (ML) from the midline (DMS: AP: 0.7 mm; ML: +1.5 mm; and DV: −3 mm; PFC: AP: 1.93 mm; ML: +0.3 mm; and DV: −2.15 mm). To monitor the neuronal activity of MSNs during TTSCS, C57BL/6J mice were injected with 200 nL rAAV-hSyn-CaMKII-GCaMP6s -WPRE-hGH-pA (Cat# PT-0110, BrainVTA., China.) and an optical fiber (200 μm OD, 0.37 NA) was placed 100 µm above the injection site. To monitor the neuronal activity of D1 or D2 MSNs during TTSCS, D1-Cre or A2a-Cre mice were injected with 200 nL rAAV-hSyn-DIO-GCaMP6s-WPRE-pA (Cat# BC-0238, BrainCase., China) and an optical fiber (200 μm OD, 0.37 NA) was placed 100 µm above the injection site. For optogenetic manipulation of DMS D2 MSNs, rAAV2/9-EF1*α*-DIO-hChR2(H134R)-EYFP-WPRE (Cat#: PT-0001, BrainVTA., China) was injected bilaterally into the DMS at a volume of 200 nL of A2a Cre mice and optic fiber were implanted into bilateral DMS. Transsynaptic anterograde tracing was used to specifically activate the postsynaptic D1 or D2 MSNs subtypes of PFC-DMS pathway. rAAV2/1-hSyn-FLP-WPRE (Cat# BC-0171, BrainCase., China) was injected into PFC at a volume of 120 nl, and rAAV2/9-hSyn-Con/Fon-hChR2(H134R)-EYFP-WPRE (Cat#: PT-0065, BrainVTA., China) was injected into DMS at a volume of 200 nl. An optical fiber (200 μm OD, 0.37 NA) was implanted into the DMS.

### Conditioned flight paradigm

Two different contexts were used. Context A (low-threat context, 200D × 400H mm) consisted of a clear cylindrical chamber with a smooth floor, while Context B (high-threat context, 200 L × 200 W × 300H mm) consisted of a square enclosure with an electrical grid floor used to deliver alternating current footshocks and a programmable audio generator for auditory stimulus (Shanghai Vanbi Intelligent Technology Co., Ltd.).

For the SCS conditioned flight paradigm test, we used the methods previously described [7, 8]. Briefly, on day 1, after 4-min habituation in the context A environment, an auditory SCS consisting of a 10 s pure tone (500 ms, 7.5 kHz pips at 1 Hz, 75 dB) and 10 s of white noise (500 ms, pips at 1 Hz, random and composed of frequencies ranging from 1 to 20 kHz, 75 dB) was delivered four times with a 60 s ITI (inter-trial interval, ITI). On days 2 and 3, the mice were conditioned five times in context B after a 4-min habituation period by pairing the SCS with the US (footshock 0.9 mA, 1 s) at an average pseudorandom ITI of 180 s, the shock was applied at the end of the last pip. On day 4, the mice were placed back in context A and, after a 4-min habituation period, were presented with the SCS four times at an ITI of 60 s.

For the TTSCS conditioned flight paradigm test, we replace the 10 s pure tone and 10 s white noise stimuli in the SCS with two pure tones (HT, high frequency tone, 500 ms, 4 kHz pips at 1 Hz, 75 dB; HT, low frequency tone, 500 ms, 400 Hz pips at 1 Hz, 75 dB) of different frequencies. And for the reverse TTSCS conditioned flight paradigm test, we switched the order of HT and LT.

### Quantification of defensive behavior

All behavioral assessments were video recorded using Tracking Master V4.0 (Shanghai Vanbi Intelligent Technology Co., Ltd.). The body of each animal was extracted from the background, and the center of gravity was used to calculate its speed. Freezing was defined as a complete cessation of movement for at least 1 s and was automatically scored using a frame-by-frame analysis of pixel changes. The flight score was calculated by dividing the average speed during exposure to each CS by the average speed during the 10 s before CS onset (speed CS/speed pre), and then adding 1 point for each jump. The first CS is taken for 10 s, and the second CS is taken for 9 s to exclude the impact of the footshock at the last pip. Escape jumping was scored manually from video files by a trained observer.

For the quantification of defensive behavior in the mice with synchronized recording of neuronal calcium activity, we tracked the mouse’s trajectory at a higher frame rate (Noldus Ethovision XT 11) to match the sampling rate of the calcium signals. We extracted the speed during the 10 s before SCS onset and the speed during the SCS period.

### Fiber photometry recording and data analysis

We used a fiber photometry system (Thinker Tech) to record the fluorescence signals in freely moving mice. Purple (405 nm) light-emitting diode (LED) light was band-pass filtered (405/10 nm, model 65133, Edmund Optics), blue (473 nm) LED light (Cree LED) was band-pass filtered (470/25 nm, model 65144, Edmund Optics). Light beam was reflected by a dichroic mirror (model 67079, 495-nm long pass, Edmund Optics) and passed through a multi-band filter (model 87-282, Edmund Optics), before being focused using a 20× objective lens (Olympus) and funneled into an optical fiber, and the light was guided between the commutator and the implanted optical fiber cannula. The laser power at the tip of the optical fiber was adjusted to 50 μW (470 nm) to minimize photobleaching. The 405-nm signal (20 μW) was further used to correct movement artifacts. The signal elicited by the 410-nm LED was linearly scaled using least-squares regression to minimize the difference between signals elicited by the 410- and 470-nm LEDs.

For data analysis, raw data were processed with custom MATLAB scripts (Thinker Tech, Nanjing Biotech) to correct for photobleaching and movement artefacts by subtracting the scaled 410-nm signal from the 470-nm signal. Then, the conditioned stimulus (CS)-evoked response was quantified. A baseline period (−2 to 0 s relative to CS onset) was defined, and the fluorescence change was converted to a Z-score. Finally, the results were visualized as Z-score heatmaps and average plots (with shaded SEM) and statistically compared based on the area under the curve (AUC) for each trial.

### Optogenetic manipulation

For optogenetic stimulation, during the laser on trials, blue light stimulation (470 nm, 20 HZ, 5 ms, 5 mW at the fiber tip) was delivered starting 2 s before the onset of the auditory stimulus and lasted until the stimulus ended, while the behavior of the mice was observed simultaneously.

### Quantification and statistical analysis

All data were transferred to SPSS 21.0 software (IBM, http://www.spss.com.cn) for analysis and to OriginPro 2021 software (OriginLab, https://www.originlab.com) for graphing. Normality testing was performed by the Shapiro–Wilk test. The homogeneity of variance test was performed by Levene test. Data that met these two conditions were analyzed using a two-tailed unpaired t-test or one-factor ANOVA and Bonferroni correction for post hoc test. Datasets that were not normally distributed were analyzed with a Mann-Whitney U test or Kruskal-Wallis H test and Nemenyi multiple comparisons test. For the paired data, datasets that were normally distributed were analyzed with Two-tailed t-test, datasets that were not normally distributed were analyzed with Mann-Whitney U test. More statistical methods are provided in Table S1. The significance levels for all tests were set at *p < 0.05, **p < 0.01, and ***p < 0.001.

## Results

### The switching between freezing and flight induced by TTSCS

**Fig. 1:**
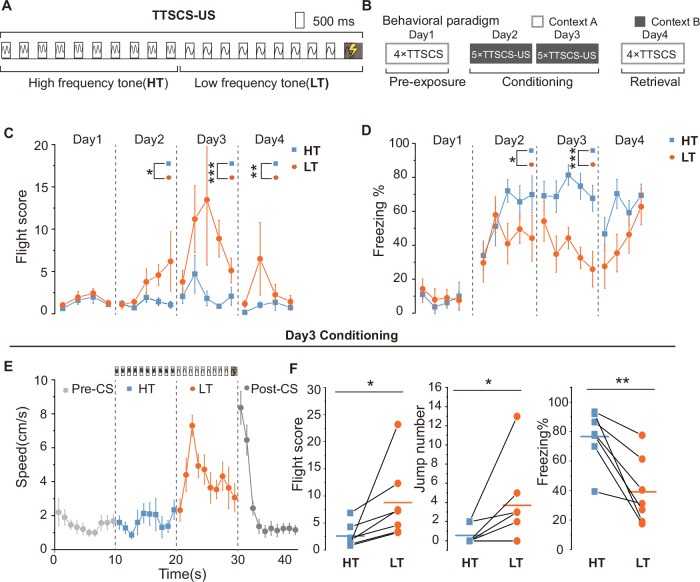
Two-tone serial compound stimulus Pavlovian conditioning paradigm. **A** The conditioning stimulus is a two-tone serial compound stimulus (TTSCS) paired with a 1s footshock during conditioning. **B** Schematic of the behavioral protocol used to induce conditioned flight behavior. **C** Comparison of the flight score between HT (blue) and LT (red) across sessions. Day1, *Friedman’s M test* (*n*=7 mice): *χ*^*2*^=3.571, *df*=1, *p*=0.059; Day2, *Friedman’s M test* (*n*=7 mice): *χ*^*2*^=6.429, *df*=1, *p*=0.011; Day3, *Friedman’s M test* (*n*=7 mice): *χ*^*2*^=24.029, *df*=1, *p*=0.000; Day4, *Friedman’s M test* (*n*=7 mice): *χ*^*2*^=9.143, *df*=1, *p*=0.002. **D** Comparison of the freezing between HT (blue) and LT (red) across sessions. Day1, *Friedman’s M test* (n=7 mice): *χ*^*2*^=0.067, *df*=1, *p*=0.796; Day2, *Friedman’s M test* (*n*=7 mice): *χ*^*2*^=5.765, *df*=1, *p*=0.016; Day3, *Friedman’s M test* (n=7 mice): *χ*^*2*^=24.029, *df*=1, *p*=0.000; Day4, *Friedman’s M test* (*n*=7 mice): *χ*^*2*^=2.462, *df*=1, *p*=0.117. **E** Average speed curve (mean±s.e.m., *n*=7 mice) on day 3; note the increase in speed during the onset of LT. **F** Left, flight scores on day 3*, Wilcoxon signed-rank test* (n=7 mice): *Z*=−2.366, *p*=0.018. Middle, the number of jump escape responses on day 3, *Wilcoxon signed-rank test* (*n*=7 mice): *Z*=−2.023, *p*=0.043. Right, freezing behavior on day 3, *two-tailed paired t-test* (*n*=7 mice): *t*=4.353, *df*=6, *p*=0.005.

To investigate the dynamic switching between freezing and flight behaviors, we employed a two-tone serial compound stimulus (TTSCS) in mice. Specifically, we paired a TTSCS, consisting of a high-frequency tone (HT, 4 kHz, 500 ms, 75 dB) followed by a low-frequency tone (LT, 400 Hz, 500 ms, 75 dB), with a footshock (US, 0.9 mA) (Fig. 1A, B, Table S2). This paradigm is based on the SCS previously described [7], but uses two distinct pure tones instead of a tone and white noise. After conditioning, mice exhibited different defensive responses during TTSCS on day 3 (Fig. 1C–E, Video S1). We quantified the active defensive response (flight) by measuring increases in speed (Fig. 1E) and the number of escape jumps (Fig. 1F, **middle**). We found that mice showed significantly higher flight scores and jump numbers during exposure to the LT compared to the HT (Fig. 1F, **left and middle**). Conversely, freezing behavior was more pronounced during HT exposure than LT exposure (Fig. 1F, **right**). These results indicate that the second tone, which is temporally closer to the US, elicited a stronger flight response. To further test this hypothesis, we reversed the order of the two pure tones (Fig. S2A). Consistent with our initial findings, mice exhibited more flight behavior during exposure to the tone that was closer to the US (Fig. S2B–F). We also replicated these results using the classic SCS paradigm (Fig. S1A–F), as previously reported [7]. Collectively, these results suggest that the TTSCS paradigm can be used to detect the switching of defensive responses to an approaching threat.

### Mice exhibit faster and stronger defensive responses as the threat approaches

**Fig. 2:**
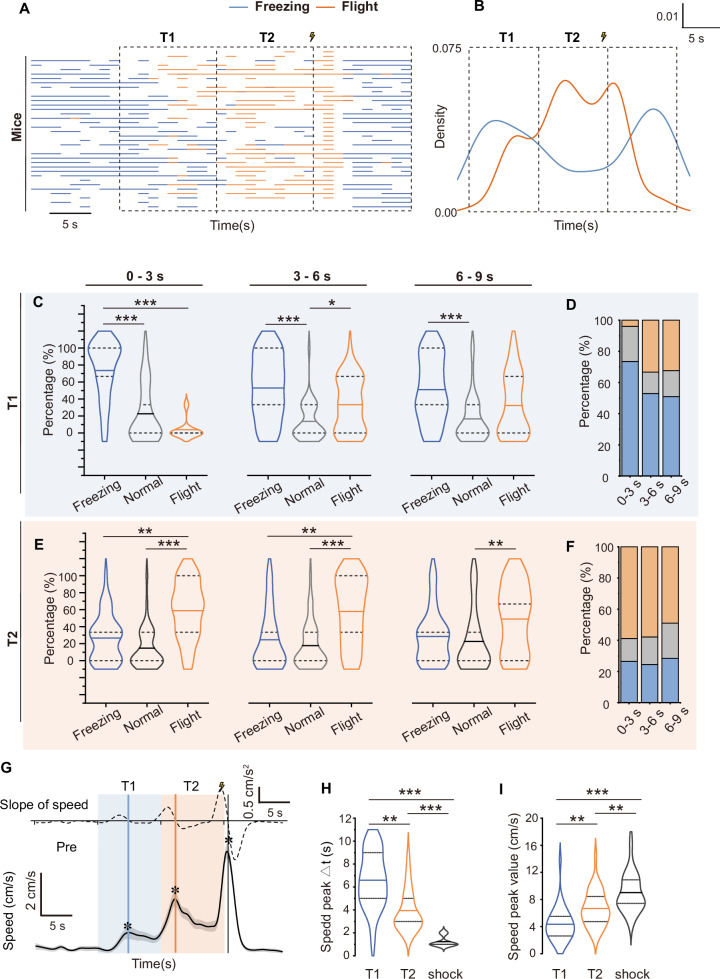
The distribution of freezing and flight responses. **A** The type of defensive response exhibited by each mouse (n=34 mice). **B** The density distribution of the two defensive responses (red: flight; blue: freezing). **C, D** Comparison of the proportion of different types of defensive responses during three intervals (0–3s, 3–6s, 6–9s) while the T1 stimulus is being presented. Left **C,** *Kruskal-Wallis H test* and *Nemenyi multiple comparisons test* for post hoc test (*n*=34 mice in the freezing, flight, and normal group): *χ*^2^=53.609, *df*=2, *p*=0.000; *χ*^2^_freezing&normal_=33.412, *p*_freezing&normal_=0.000; *χ*^2^_flight&normal_=13.294, *p*_flight&normal_=0.129; *χ*^2^_freezing&flight_=46.706, *p*_freezing&flight_=0.000. Middle **C,** *Kruskal-Wallis H test* and *Nemenyi multiple comparisons test* for post hoc test (*n*=34 mice in the freezing, flight, and normal group): *χ*^2^=21.592, *df*=2, *p*=0.000; *χ*^2^_freezing&normal_=31.559, *p*_freezing&normal_=0.000; *χ*^2^_flight&normal_=−17.324, *p*_flight&normal_=0.033; *χ*^2^_freezing&flight_=14.235, *p*_freezing&flight_=0.109; Right **C,** *Kruskal-Wallis H test* and *Nemenyi multiple comparisons test* for post hoc test (*n*=34 mice in the freezing, flight, and normal group): *χ*^2^=16.021, *df*=2, *p*=0.000; *χ*^2^_freezing&normal_=27.074, *p*_freezing&normal_=0.000; *χ*^2^_flight&normal_=14.662, *p*_flight&normal_=0.091; *χ*^2^_freezing&flight_=−12.412, *p*_freezing&flight_=0.200. **E, F** Comparison of the proportion of different types of defensive responses during three intervals (0–3s, 3–6s, 6–9s) while the T2 stimulus is being presented. Left (E), *Kruskal-Wallis H test* and *Nemenyi multiple comparisons test* for post hoc test (n=34 mice in the freezing, flight, and normal group): *χ*^2^=30.913, *df*=2, *p*=0.000; *χ*^2^_freezing&normal_=11.588, *p*_freezing&normal_=0.266; *χ*^2^_flight&normal_=−36.985, *p*_flight&normal_=0.000; *χ*^2^_freezing&flight_=−25.397, *p*_freezing&flight_=0.001. Middle (E), *Kruskal-Wallis H test* and *Nemenyi multiple comparisons test* for post hoc test (*n*=34 mice in the freezing, flight, and normal group): *χ*=21.020, *df*=2, *p*=0.000; *χ*^2^_freezing&normal_=4.721, *p*_freezing&normal_=1.000; *χ*^2^_flight&normal_=−28.809, *p*_flight&normal_=0.000; *χ*^2^_freezing&flight_=−24.088, *p*_freezing&flight_=0.001. Right (E), *Kruskal-Wallis H test* and *Nemenyi multiple comparisons test* for post hoc test (n=34 mice in the freezing, flight, and normal group): *χ*^2^=9.813, *df*=2, *p*=0.007; *χ*^2^_freezing&normal_=6.971, *p*_freezing&normal_=0.909; *χ*^2^_flight&normal_=−20.824, *p*_flight&normal_=0.006; *χ*^2^_freezing&flight_=−13.853, *p*_freezing&flight_=0.122. **G** The average velocity traces (bottom) and velocity change rate traces (top) of all mice. **H** Comparison of the latency of the velocity peaks caused by the increase in speed in response to three different types of stimuli (T1, T2 and foot-shock). *Kruskal-Wallis H test* and *Nemenyi multiple comparisons test* for post hoc test (n=34 mice in the freezing, flight, and normal group): *χ*^2^=73.036, *df*=2, *p*=0.000; *χ*^2^_T1&T2_=21.294, *p*_T1&T2_=0.008; *χ*^2^_T1&shock_=59.662, *p*_T1&shock_=0.000; *χ*^2^_T2&shock_=38.368, *p*_T2&shock_=0.000. **I** Comparison of the amplitude of the velocity peaks caused by the increase in speed in response to three different types of stimuli (T1, T2 and foot-shock). *Kruskal-Wallis H test* and *Nemenyi multiple comparisons test* for post hoc test (n=34 mice in the freezing, flight, and normal group): *χ*^2^=41.363, *df*=2, *p*=0.000; *χ*^2^_T1&T2_=−24.647, *p*_T1&T2_=0.002; *χ*^2^_T1&shock_=−46.118, *p*_T1&shock_=0.000; *χ*^2^_T2&shock_=−21.471, *p*_T2&shock_=0.008.

To further investigate the characteristics of the switch in defensive responses as the threat approaches. We defined the first auditory stimulus in the SCS composed of different types of stimuli as T1 and the subsequent second stimulus as T2. We established a velocity-based classifier to determine the state of the mice, following the definitions of freezing and flight reported in previous studies (Fig. 2A). Our analysis revealed that during the entire SCS period, the proportion of freezing responses gradually decreased, while the proportion of flight responses progressively increased (Fig. 2A, B). Interestingly, the proportion of freezing increased at the T1 stage and then smoothly decreased, whereas the proportion of flight exhibited peaks with different latencies during the T1, T2, and foot-shock stimulation stages (Fig. 2B). To better understand the temporal dynamics of these two behaviors, we divided T1 and T2 into three segments of 3s each and calculated the proportion of each behavior within each time segment. We found that freezing predominantly occurred within the 0–3s, after which the proportion of flight began to increase (Fig. 2C, D) during T1. Flight behavior accounted for a relatively large proportion in all three intervals during T2 (Fig. 2E, F). Given that flight is determined by velocity, we averaged the velocity of all mice during the SCS and we found that following exposure to the three different levels of threat stimuli—T1, T2, and foot-shock, mice all exhibited increased velocity, corresponding to three peaks of different shapes (Fig. 2G). After calculating the latency and amplitude of these three peaks, we found that as the threat approached, the latency of the peaks gradually decreased (Fig. 2H), while their amplitude progressively increased (Fig. 2I). In summary, mice flexibly exhibit defensive responses when facing threat, as the threat stimulus approached more closely, mice tended to exhibit more escape behaviors with shorter latencies and larger magnitudes.

### D1 and D2 MSNs in the DMS dynamically participate in the defensive responses

**Fig. 3:**
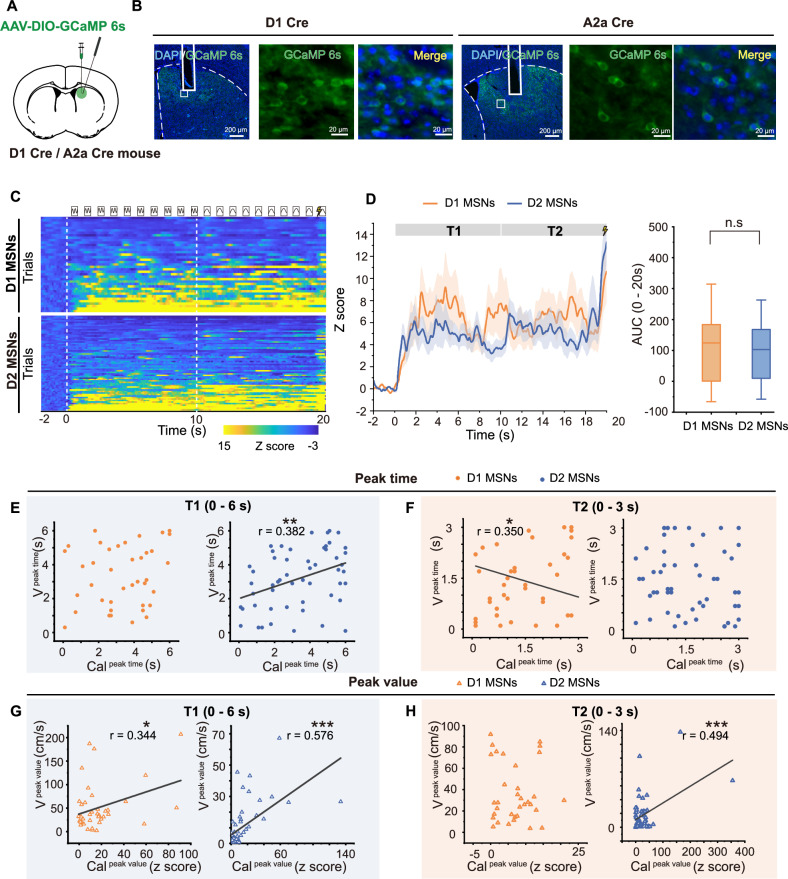
Activation of D1 and D2 MSNs in the DMS during defensive behavior. **A** Schematic representation of the setup of fiber photometry to record calcium activity from D1 or D2 MSNs infected with rAAV-hSyn-DIO-GCaMP6s virus in the D1 Cre or A2a Cre mice. **B** Expression of GCaMP6s (green) and placement of the fiber optics were verified postmortem in the D1 Cre mice (left) and A2a Cre mice (right). Scale bar, 200µm for the big image and 20µm for blown-up images. **C** All results of calcium activity in D1 MSNs (top) and D2 MSNs (bottom) in the DMS during flight conditioning on day 3. **D** Averaged traces of calcium activity (left) in D1 MSNs (red) and D2 MSNs (blue) and the area under the curve (AUC) of calcium changes (right) during flight conditioning on day 3. The AUC (area under the curve) is commonly employed to quantify the amplitude of neuronal calcium dynamics over a specific time window. *Mann-Whitney U test* (n=39 trials from 6 mice in D1 MSNs group and n=64 trials from 11 mice in D2 MSNs group): *Z*=−0.462, *p*=0.644. **E** Scatter plot showing the relationship between the calcium signal peak time of MSNs (D1: left, red; D2: right, blue) and velocity peak time. Left, *Pearson correlation coefficient* (*r*) = 0.177, *p*=0.280. Right, *Pearson correlation coefficient* (*r*) = 0.382, *p*=0.005. Data points represent individual measurements, and the solid line indicates the linear regression fit. **F** Scatter plot showing the relationship between the calcium signal peak time of MSNs (D1: left, red; D2: right, blue) and velocity peak time. Left, *Pearson correlation coefficient* (*r*) = 0.350, *p*=0.029. Right, *Pearson correlation coefficient* (*r*)=−0.094, *p*=0.503. Data points represent individual measurements, and the solid line indicates the linear regression fit. **G** Scatter plot showing the relationship between the calcium signal peak time of MSNs (D1: left, red; D2: right, blue) and velocity peak time. Left, *Pearson correlation coefficient* (*r*) = 0.344, *p*=0.032. Right, *Pearson correlation coefficient* (*r*) = 0.576, *p*=0.000. Data points represent individual measurements, and the solid line indicates the linear regression fit. **H** Scatter plot showing the relationship between the calcium signal peak time of MSNs (D1: left, red; D2: right, blue) and velocity peak time. Left, *Pearson correlation coefficient* (*r*)=−0.194, *p*=0.236. Right, *Pearson correlation coefficient* (*r*) = 0.494, *p*=0.000. Data points represent individual measurements, and the solid line indicates the linear regression fit.

Previous studies have indicated the involvement of the DMS in modulation of response timing and action initiation [17–22]. We first examined the calcium activity changes of all MSNs in the DMS during the TTSCS paradigm and found that MSNs exhibited decreased calcium activity throughout the entire SCS stimulation period (Fig. S3). To determine whether D1 and D2 MSNs in the DMS are involved in defensive responses during the TTSCS paradigm, we injected rAAV-DIO-GCaMP6s into the DMS of D1-Cre and A2a-Cre mice to record calcium activity (Fig. 3A). Viral expression and fiber placement were verified (Fig. 3B). The mice with inaccurate sites were excluded.

To quantitatively assess calcium signal changes, we established a baseline period (−2 to 0s relative to TTSCS onset) and measured calcium activity within 20s after SCS onset (Fig. 3C). Using the area under the curve (AUC) as an indicator, we found the activation levels of D1 and D2 MSNs did not show significant differences (Fig. 3D). A plausible explanation for the decreased overall activity in Fig. S3 versus the increased subtype-specific activity in Fig. 3 is that both D1 and D2 MSNs are critically engaged during defensive responses. Their antagonistic functions, coupled with potential temporal or spatial non-synchronicity in their activation patterns, could result in a net decrease in the bulk averaged signal. Given that defensive responses can vary among mice within the same behavioral paradigm, we used velocity as an index of defensive responses and performed joint analysis with calcium signals sampled at the same rate. We found that during the T1 stimulus, the latency to peak velocity was highly correlated with the latency to peak calcium activity in D2 MSNs but not in D1 MSNs (Fig. 3E). Conversely, during the T2 stimulus, the latency to peak velocity was highly correlated with the latency to peak calcium activity in D1 MSNs instead of D2 MSNs (Fig. 3F). Moreover, during T1 stimulus, the amplitude of peak velocity was highly correlated with the amplitude of peak calcium activity in both D1 and D2 MSNs (Fig. 3G), and during the T2 stimulus, the amplitude of peak velocity was highly correlated with the amplitude of peak calcium activity in D2 MSNs (Fig. 3H). Although we did not detect a significant correlation between the amplitude of peak calcium activity amplitude of D1 MSNs and velocity peaks during the T2 stimulus, we observed a positive skewness in the distribution of correlation coefficients between D1 MSNs calcium activity and velocity across the entire time axis (Fig. S4A–D). This indicates that D1 calcium activity maintains a correlation with velocity throughout the SCS paradigm. These results suggest that MSNs in the DMS are involved in defensive responses when threats approaching. Specifically, as threat intensity escalates, the latency of defensive responses shifts from being associated with D2 neuron activity to being correlated with D1 neuron activity, while the amplitude of the defensive responses involves both D1 and D2 neurons.

### PFC-DMS^D1^ pathway mediate the faster defensive responses as the threat approaches

**Fig. 4:**
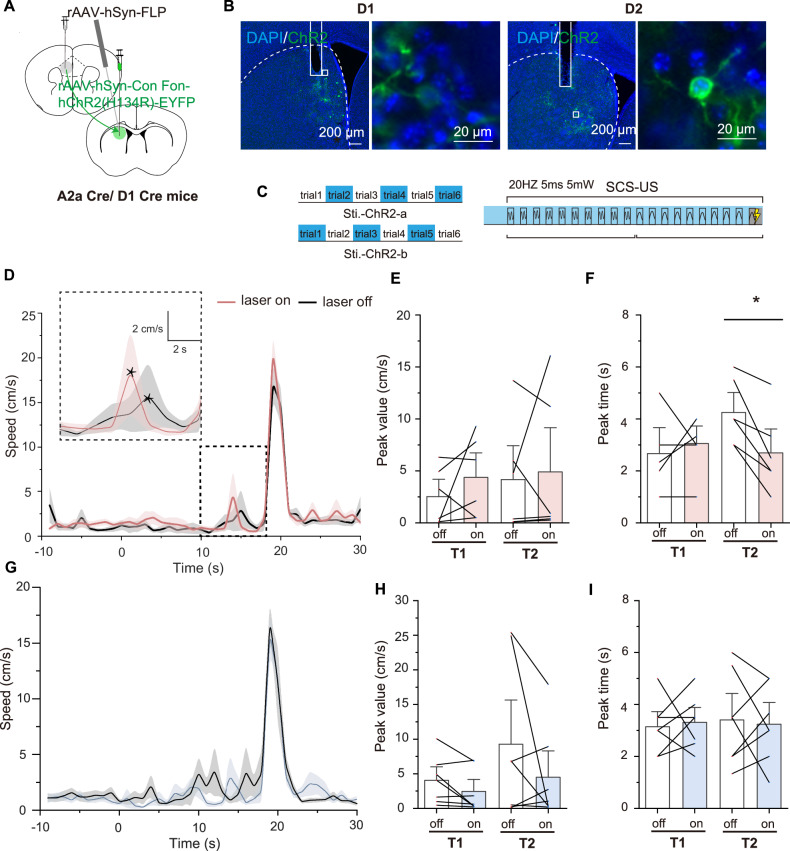
Activation of PFC-DMS pathway in the DMS during defensive behavior. **A** Schematics for optogenetics manipulation. Targeted functional activation of the D1 and D2 MSNs in the DMS that specifically receive inputs from the PFC was achieved by injection of rAAV-hSyn-FLP into the PFC, rAAV-hSyn-ConFon-hChR2(H134R)-EYFP into the DMS, and implantation of an optical fiber above the DMS in D1-Cre or A2a-Cre mice. **B** Expression of ChR2 (green) and placement of the fiber optics were verified postmortem in the DMS of D1 Cre mice (left) and A2a Cre mice (right). Scale bar, 200µm for the big image and 20µm for blown-up images. **C** sequence of the TTSCS test and the parameters of blue laser light. **D** Average speed over time (mean ± s.e.m., n=6 mice) on day 3, under laser-off and laser-on conditions, showing that activation of PFC-DMS^D1^ during T2 elicits a faster defensive response. **E** Comparison of the Peak value (cm/s) between off (white) and on (red) across sessions. (T1: left; T2: right). Left, *two-tailed paired t-test* (*n*=6 mice): *t*=−*1.108, df*=*5, p*=*0.318*. Right, *two-tailed paired t-test* (*n*=6 mice): *t*=−*0.322, df*=*5, p*=*0.761*. **F** Comparison of the Peak value (cm/s) between off (white) and on (red) across sessions. (T1: left; T2: right). Left, *two-tailed paired t-test* (*n*=5 mice): *t*=−*0.302, df*=*4, p*=*0.778*. Right, *two-tailed paired t-test* (n=6 mice): *t*=*4.076, df*=*5, p*=*0.010*. **G** Average speed over time (mean ± s.e.m., *n*=7 mice) on day 3, under laser-off and laser-on conditions. **H** Comparison of the Peak value (cm/s) between off (white) and on (blue) across sessions. (T1: left; T2: right). Left, *two-tailed paired t-test* (n=7 mice): *t*=*1.996, df*=*6, p*=*0.093*. Right, *two-tailed paired t-test* (*n*=7mice): *t*=*1.303, df*=*6, p*=*0.240*. **I** Comparison of the Peak value (cm/s) between off (white) and on (blue) across sessions. (T1: left; T2: right). Left, *two-tailed paired t-test* (*n*=7 mice): *t*=−*0.312, df*=*6, p*=*0.766*. Right, *two-tailed paired t-test* (*n*=7 mice): *t*=*0.243, df*=*6, p*=*0.816*.

We conducted optogenetic experiments targeting D2 MSNs in the DMS (Fig. S5A–C), comparing to the light-off period, activation of D2 MSNs resulted in a robust reduction in mouse locomotor activity, which was observed in both the adaptive phase (Video S2) and the TTSCS behavioral paradigm (Fig. S5D-M, Video S3). Given that direct manipulation of MSNs in the DMS can profoundly affect general locomotion, we selectively activated D1 or D2 MSNs in the DMS that specifically receive inputs from the PFC to investigate their involvement in action initiation during defensive responses (Fig. 4A, B). Mice were randomly assigned to two groups that received the laser stimulation in different sequences (Sti.-ChR2-a or Sti.-ChR2-b) to counterbalance the order effects (Fig. 4C, **left**). In the laser on period, the auditory stimulus was paired with blue laser to activate the specific postsynaptic D1 or D2 MSNs subtypes of PFC-DMS pathway (Fig. 4C, **right**). Activation of D1 MSNs subtypes of PFC-DMS pathway did not affect the peak or average speed of mice in response to either the T1 or T2 (Fig. 4D, E), whereas it significantly shortened the latency to the peak speed upon T2 (Fig. 4F, Video S4). This result suggests that PFC-DMS^D1^ pathway activation prompts faster defensive responses to an elevated threat. Activation of D2 MSNs subtypes of PFC-DMS pathway resulted in no significant alterations in the average speed, peak speed, or the latency to peak speed during either T1 or T2 stimulation (Fig. 4G–I). In summary, our results suggested that PFC-DMS^D1^ pathway can regulate response timing during defensive responses; its activation enables mice to execute faster defensive responses when confronted with an approaching threat stimulus.

## Discussion

The present study successfully developed the TTSCS paradigm and demonstrated that increasing threat levels elicit faster and more intense defensive responses. This dynamic adjustment to threat stimuli appears to be linked to the engagement of D1 and D2 neurons in the DMS. Specifically, as threat intensity escalates, the latency of defensive responses shifts from being associated with D2 neuron activity to correlating with D1 neuron activity, while the amplitude of the defensive responses involves both D1 and D2 neurons. Following functional validation through manipulation of the PFC–DMS circuit, we found that activating the PFC-innervated direct pathway in the DMS significantly shortened the response latency of mice to heightened threat stimuli, enabling faster defensive reactions.

Although sound-conditioned stimuli (SCS) is a classical paradigm for exploring the conditioned defensive response, the underlying mechanisms remain debated. Previous studies indicate that parameters such as stimulus frequency, order, and sound pressure level can influence defensive behaviors, with white noise generally eliciting stronger physiological and behavioral responses than pure tones [30–32]. Consistently, we observed more robust defensive behaviors to white noise compared to pure tones prior to training (Fig. S1C, **Day1**; Fig. S1D, **Day1**). Moreover, a transition to flight during serial auditory conditioning has been previously interpreted as an unconditioned response to sudden stimulus changes [33]. Therefore, to minimize the potential confounding effect of stimulus salience, we designed both CS1 and CS2 as pure tones (75 dB) differing only in frequency within the TTSCS paradigm (Fig. 1A, B) and reversed their order in the reverse TTSCS paradigm (Fig. S2A, B). And we also habituated the mice to the auditory stimuli on Day 1 to reduce novelty effects. On Day 1, before footshock pairing, mice exhibited no significant defense or escape to tone presentation or its sudden change (Fig. 1C, D, **Day 1**). It suggests that our TTSCS paradigm indeed minimizes the effect of sound parameters on conditioned defensive responses. Interestingly, we observed a negative AUC on Day 1 (Fig. S3). We suspect this may be attributed to a weak response to the novel stimulus. Once the mice adapted to the sound stimuli, no significant defensive responses or notable changes in calcium signals were observed. After conditioning on Day 2, however, mice gradually differentiated their defensive responses based on tone identity. On Day 3 of testing, TTSCS captured threat-dependent modulation of defensive responses. We propose that the shift between response types is better explained by the proximity of the threat rather than by a direct readout of Pavlovian conditioning. This is consistent with previous observations that mice switch defensive responses to naturalistic predator threats based on predator proximity [34–36]. Indeed, mice freeze to avoid detection when a predator is distant but switch to flight when the predator approaches too closely. This may reflect the transition from fear to panic. Our paradigm thus demonstrates that the switching of learned defensive responses is tightly linked to threat imminence.

Interestingly, we also found that as the threat stimulus approached more closely, mice exhibited more escape behaviors with shorter latencies and larger magnitudes of response, which associated with the dynamic responses of D1 and D2 MSNs in the DMS. Specifically, in the switching of defensive responses, D2 MSNs mediate freezing (a conservative defensive response), while D1 MSNs involve in flight (a riskier defensive response). The DMS is known to plays a crucial role in action initiation [12, 13, 16, 37] and complex temporal processing [38]. The direct pathway activation disinhibits brainstem motor structures, and thalamic nuclei targeting the motor cortex, promoting movement. In contrast, the indirect pathway activates basal ganglia output nuclei and thus inhibits movements [39, 40]. In this study, we conducted optogenetic activation targeting D2 MSNs in the DMS (Fig. S5A–C), and found a robust reduction in mouse locomotor activity (Video S2, Fig. S4D–M, Video S3). This suggests that MSN neurons in the DMS may predominantly regulate arousal state as well as the initiation and dynamic modulation of action [41, 42]. To investigate the involvement of the DMS in action initiation during defensive responses, and considering that the DMS receives substantial and highly overlapping inputs from the PFC [23, 24], which regulate defensive behaviors [43, 44], we selectively activated D1 or D2 MSNs in the DMS that specifically receive inputs from the PFC. We found that PFC-DMS^D1^ pathway regulates response timing during defensive responses, its activation enables mice to execute defensive responses more rapidly when confronted with an approaching threat stimulus. In conclusion, D1and D2 MSNs in the DMS likely act in an antagonistic manner to regulate defensive behaviors. Based on the function of the DMS [17–22], the activity of MSNs we observed likely reflects dynamic motor and arousal-related processes rather than categorical threat encoding. However, these arousal-related processes can also modulate the response to threat [8, 9]. And furthermore, the PFC projection specifically to the direct pathway within the DMS mediates the shift in defensive responses to an approaching threat. Given that the critical role of dysregulated dopaminergic modulation of striatum in the schizophrenia and anxiety [41, 42]. The identification of a circuit mechanism that precisely regulates defensive responses, dissociated from general locomotion, offers a potential avenue for developing new treatments for disorders involving abnormal defensive reactions. For example, combining region-specific Transcranial Magnetic Stimulation (TMS) with D1 or D2 receptor agonists or antagonists could be used to treat schizophrenia or panic disorder, leading to more accurate therapeutic outcomes and reduced side effects.

However, our study has certain limitations. Due to limitations of the viral tools, we were unable to simultaneously record calcium signals from both D1 and D2 MSNs in the same mouse. While we identified a correlation between MSNs activity in the DMS and the action initiation processes underlying defensive responses, we did not establish their causal roles. Previous studies have shown that sex hormones, along with other peripheral signals such as cytokines and metabolic hormones, play a significant role in shaping defensive responses [1, 45]. However, in the present study, we only focused exclusively on defensive behaviors in male mice, and did not include an investigation of female subjects.

## Supplementary information

Supplementary figure legends, table and video title

Supplementary Table 1

Supplementary Table 2

Supplementary Figure 1

Supplementary Figure 2

Supplementary Figure 3

Supplementary Figure 4

Supplementary Figure 5

Supplementary Video 1

Supplementary Video 2

Supplementary Video 3

Supplementary Video 4

## Data Availability

All data supporting the findings of this study are available within the article and its Supplementary Information files.
