# Supplementary material for: A corticostriatal circuit mediates the switching of defensive responses to an approaching threat
Source: Transl Psychiatry. 2026 May 20;16:357. doi: 10.1038/s41398-026-04105-3 (PMC13365514; doi:10.1038/s41398-026-04105-3)
Supplement: Supplementary file 1 — Supplementary figure legends, table and video title [file 41398_2026_4105_MOESM1_ESM.docx]

**Figure S1. SCS Pavlovian conditioning paradigm**

(A) The conditioning stimulus is a SCS paired with a 1s footshock during conditioning.

(B) Schematic of the behavioral protocol used to induce conditioned flight behavior.

(C) Comparison of the flight score between tone (blue) and white noise (red) across sessions. Day1, *Friedman's M test* (n = 11 mice): *χ^2^* = 9.091, *df* = 1, *p* = 0.003; Day2, *Friedman's M test* (n = 11 mice): *χ^2^* = 6.564, *df* = 1, *p* = 0.010; Day3, *Friedman's M test* (n = 12 mice): *χ^2^* = 13.067, *df* = 1, *p* = 0.000; Day4, *Friedman's M test* (n = 12 mice): *χ^2^* = 5.333, *df* = 1, *p* = 0.021.

(D) Comparison of the freezing between tone (blue) and white noise (red) across sessions. Day1, *Friedman's M test* (n = 10 mice): *χ^2^* = 16.892, *df* = 1, *p* = 0. 000; Day2, *Friedman's M test* (n = 12 mice): *χ^2^* = 0.067, *df* = 1, *p* = 0.796; Day3, *Friedman's M test* (n = 12 mice): *χ^2^* = 4.267, *df* = 1, *p* = 0.039; Day4, *Friedman's M test* (n = 12 mice): *χ^2^* = 3.596, *df* = 1, *p* = 0.058.

(E) Average speed curve (mean ± s.e.m., n = 7 mice) on day 3; note the increase in speed during the onset of white noise.

(F) Left, flight scores on day 3*, Wilcoxon signed-rank test* (n = 12 mice): *Z =* -2.824, *p =* 0.005. Right, freezing behavior on day 3, *Wilcoxon signed-rank test* (n = 12 mice): *Z =* -2.434, *p =* 0.015.

**Figure S2. Reverse TTSCS Pavlovian conditioning paradigm**

(A) The conditioning stimulus is a reverse TTSCS paired with a 1s footshock during conditioning.

(B) Comparison of the flight score between HT (red) and LT (blue) across sessions. Day1, *Friedman's M test* (n = 16 mice): *χ^2^* = 0.342, *df* = 1, *p* = 0.558; Day2, *Friedman's M test* (n = 16 mice): *χ^2^* = 2.909, *df* = 1, *p* = 0.088; Day3, *Friedman's M test* (n = 16 mice): *χ^2^* = 29.225, *df* = 1, *p* = 0.000; Day4, *Friedman's M test* (n = 16 mice): *χ^2^* = 4.056, *df* = 1, *p* = 0.044.

(C) Comparison of the freezing between HT (red) and LT (blue) across sessions. Day1, *Friedman's M test* (n = 16 mice): *χ^2^* = 33.882, *df* = 1, *p* = 0.000; Day2, *Friedman's M test* (n = 16 mice): *χ^2^* = 20.045, *df* = 1, *p* = 0.000; Day3, *Friedman's M test* (n = 16 mice): *χ^2^* = 0.551, *df* = 1, *p* = 0.458; Day4, *Friedman's M test* (n = 16 mice): *χ^2^* = 33.136, *df* = 1, *p* = 0.000.

(D) Average speed curve (mean ± s.e.m., n = 16 mice) on day 3; note the increase in speed during the onset of HT.

(E) Flight scores on day 3*, Wilcoxon signed-rank test* (n = 16 mice): *Z =* -3.942, *p =* 0.000.

(F) Freezing behavior on day 3, *Wilcoxon signed-rank test* (n = 16 mice): *t =* -1.466, *p =* 0.143.

**Figure S3. The involvement of MSNs during TTSCS Pavlovian conditioning paradigm**

(A) Schematic representation of the setup of fiber photometry to record calcium activity from MSNs infected with rAAV-CaMKII--GCaMP6s virus in the C57/BL6 mice.

(B) Expression of GCaMP6s and placement of the fiber optics were verified post-mortem. 200 μm for the big image and 20 μm for blown-up images.

(C) All results of calcium activity (left) and averaged traces of each mouse (right) in MSNs in the DMS on day 1.

(D) Averaged traces of calcium activity in MSNs (left) and the area under the curve (AUC) of calcium changes (right) on day 1. Two-tailed unpaired t-test (n = 6 mice): *t =* 2.778, *df* = 5, *p =* 0.039; Two-tailed unpaired t-test (n = 6 mice): *t =* 0.548, *df* = 5, *p =* 0.607.

(E) All results of calcium activity (left) and averaged traces of each mouse (right) in MSNs in the DMS on day 3.

(F) Averaged traces of calcium activity in MSNs (left) and the area under the curve (AUC) of calcium changes (right) on day 3. Two-tailed unpaired t-test (n = 6 mice): *t =* 3.140, *df* = 5, *p =* 0.026; Two-tailed unpaired t-test (n = 6 mice): *t =* 3.752, *df* = 5, *p =* 0.013.

**Figure S4. The distribution of correlation coefficients between calcium signals and velocity**

(A) The distribution of correlation coefficients between calcium signals (red: D1 MSNs; blue: D2 MSNs) and velocity in 0-3 s (top), 3-6 s (middle), and 6-10 s (bottom).

(B) Correlation coefficients between calcium signals and velocity (top) and the proportion of correlation coefficients with significant differences (bottom) in -2-0 s, 0-3 s, 3-6 s, and 6-10 s during T1.

(C) The distribution of correlation coefficients between calcium signals (red: D1 MSNs; blue: D2 MSNs) and velocity in 0-3 s (top), 3-6 s (middle), and 6-10 s (bottom).

(D) Correlation coefficients between calcium signals and velocity (top) and the proportion of correlation coefficients with significant differences (bottom) in 0-3 s, 3-6 s, and 6-10 s during T2.

**Figure S5. Activation of D2 MSNs in the DMS during defensive behavior**

(A) Expression of ChR2 (green) and placement of the fiber optics were verified postmortem in the DMS of A2a Cre mice. Scale bar, 1 mm.

(B-C) Sequence of the TTSCS test and the parameters of blue laser light.

(D) The average velocity traces of all mice during laser on period-T1 (blue) and laser off period (black).

(E) Comparison of the speed (cm/s) between off (white) and on (blue) (T1, left), and between off (T2, right). Left, *two-tailed paired t-test* (n = 3 mice): *t = 2.024, df = 2, p = 0.180.* Right, *two-tailed paired t-test* (n = 3 mice): *t = 0.748, df = 2, p = 0.532.*

(F) Comparison of the speed (cm/s) between off (white) and on (blue) (T1, left), and between off (T2, right). Left, *two-tailed paired t-test* (n = 3 mice): *t = 1.438, df = 2, p = 0.287.* Right, *two-tailed paired t-test* (n = 3 mice): *t = 1.267, df = 2, p = 0.333.*

(G) Comparison of the speed (cm/s) between off (white) and on (blue) (T1, left), and between off (T2, right). Left, *two-tailed paired t-test* (n = 3 mice): *t = 1.553, df = 2, p = 0.261.* Right, *two-tailed paired t-test* (n = 3 mice): *t = 0.163, df = 2, p = 0.885.*

(H) Comparison of the speed (cm/s) between off (white) and on (blue) (T1, left), and between off (T2, right). Left, *two-tailed paired t-test* (n = 3 mice): *t = -0.795, df = 2, p = 0.510.* Right, *two-tailed paired t-test* (n = 3 mice): *t = 3.024, df = 2, p = 0.094.*

(I) The average velocity traces of all mice during laser on period-T2 (blue) and laser off period (black).

(J) Comparison of the speed (cm/s) between off (white) and on (blue) (T1, left), and between off (T2, right). Left, *two-tailed paired t-test* (n = 3 mice): *t = 3.337, df = 2, p = 0.079.* Right, *two-tailed paired t-test* (n = 3 mice): *t = 5.038, df = 2, p = 0.037.*

(K) Comparison of the speed (cm/s) between off (white) and on (blue) (T1, left), and between off (T2, right). Left, *two-tailed paired t-test* (n = 3 mice): *t = -0.724, df = 2, p = 0.544.* Right, *two-tailed paired t-test* (n = 3 mice): *t = 2.033, df = 2, p = 0.179.*

(L) Comparison of the speed (cm/s) between off (white) and on (blue) (T1, left), and between off (T2, right). Left, *two-tailed paired t-test* (n = 3 mice): *t = 5.555, df = 2, p = 0.031.* Right, *two-tailed paired t-test* (n = 3 mice): *t = 2.118, df = 2, p = 0.168.*

(M) Comparison of the speed (cm/s) between off (white) and on (blue) (T1, left), and between off (T2, right). Left, *two-tailed paired t-test* (n = 3 mice): *t = 0.397, df = 2, p = 0.729.* Right, *two-tailed paired t-test* (n = 3 mice): *t = 0.756, df = 2, p = 0.529.*

**Table S1.** Statistical methods

**Table S2.** The comparison between SCS and TTSCS

**Video S1.** Defensive responses of mice in the TTSCS paradigm

**Video S2.** Locomotor activity was decreased following the activation of D2 MSNs in the DMS

**Video S3.** Defensive responses of mice in the TTSCS following the activation of D2 MSNs in the DMS

**Video S4.** Defensive responses of mice in the TTSCS following the activation of PFC-DMS^D1^ pathway
