# Supplementary material for: A corticostriatal circuit mediates the switching of defensive responses to an approaching threat
Source: Transl Psychiatry. 2026 May 20;16:357. doi: 10.1038/s41398-026-04105-3 (PMC13365514; doi:10.1038/s41398-026-04105-3)
Supplement: Supplementary file 2 — Supplementary Table 1 [file 41398_2026_4105_MOESM2_ESM.docx]

| **Experimental design** | **Group** | **Factor** | **Normality Test (Shapiro-Wilk)** | **Homogeneity of variance test** | | **Hypothesis test** |
| --- | --- | --- | --- | --- | --- | --- |
| Completely random design | 2 groups | 1 | P>0.05, normal distribution | Levene’s test | P>0.05, meet homogeneity of variance | Two-tailed unpaired t-test |
|  |  |  |  |  | P<0.05, not meet homogeneity of variance | Two-tailed unpaired separate variance estimation t-test |
|  |  |  | P<0.05, not normal distribution | Levene’s test | meet or not meet homogeneity of variance | Mann-Whitney U test |
| Paired design | 2 groups | 1 | P>0.05, normal distribution |  |  | Two-tailed paired t-test |
|  |  |  |  |  |  |  |
|  |  |  | P<0.05, not normal distribution |  |  | Wilcoxon signed-rank test |
| Completely random design | >2 groups | 1 | P>0.05, normal distribution | Levene’s test | P>0.05, meet homogeneity of variance | One-factor ANOVA and Bonferroni correction for post hoc test |
|  |  |  |  |  | P<0.05, not meet homogeneity of variance | Kruskal-Wallis H test and Nemenyi multiple comparisons test |
|  |  |  | P<0.05, not normal distribution | Levene’s test | meet or not meet homogeneity of variance | Kruskal-Wallis H test and Nemenyi multiple comparisons test |
| Repeated measurement design | ≥2 groups | 2 | P>0.05, normal distribution | Mauchly’s test of sphericity | P>0.05, meet homogeneity of variance | Repeated measurement ANOVA and Simple effects analysis |
|  |  |  |  |  | P<0.05, not meet homogeneity of variance | Friedman's M test and Simple effects analysis |
|  |  |  | P<0.05, not normal distribution | Mauchly’s test of sphericity | meet or not meet homogeneity of variance | Friedman's M test and Simple effects analysis |
|  |  |  |  |  |  |  |

**Table S1. Statistical methods**
