# Supplementary material for: A corticostriatal circuit mediates the switching of defensive responses to an approaching threat
Source: Transl Psychiatry. 2026 May 20;16:357. doi: 10.1038/s41398-026-04105-3 (PMC13365514; doi:10.1038/s41398-026-04105-3)
Supplement: Supplementary file 3 — Supplementary Table 2 [file 41398_2026_4105_MOESM3_ESM.docx]

**Table S2. The comparison between SCS and TTSCS**

| Conditioned flight paradigm | Day | Context | Habituation Time | Stimulus Type | Stimulus Order | Stimulus Times | Inter-Trial Interval  (ITI) | Conditioning Process |
| --- | --- | --- | --- | --- | --- | --- | --- | --- |
| SCS | Day 1 | Context A | 4 minutes | 10 s pure tone | Pure tone → White noise | 4 times | 60 s | None |
|  | Day 2 and 3 | Context B | 4 minutes | 10 s pure tone + 10 s white noise | Pure tone → White noise | 5 times | Average pseudorandom 180 s | SCS paired with footshock (0.9 mA, 1 s) at the end of the last pip |
|  | Day 4 | Context A | 4 minutes | 10 s pure tone + 10 s white noise | Pure tone → White noise | 4 times | 60 s | None |
| TTSCS | Day 1 | Context A | 4 minutes | High-frequency tone HT + Low-frequency tone LT | HT → LT (Forward) or LT → HT (Reverse) | 4 times | 60 s | None |
|  | Day 2 and 3 | Context B | 4 minutes | HT + LT or LT + HT | HT → LT (Forward) or LT → HT (Reverse) | 5 times | Average pseudorandom 180 s | TTSCS paired with footshock (0.9 mA, 1 s) at the end of the last pip |
|  | Day 4 | Context A | 4 minutes | HT + LT or LT + HT | HT → LT (Forward) or LT → HT (Reverse) | 4 times | 60 s | None |
