# Supplementary figures and images for: A corticostriatal circuit mediates the switching of defensive responses to an approaching threat
Source: Transl Psychiatry. 2026 May 20;16:357. doi: 10.1038/s41398-026-04105-3 (PMC13365514; doi:10.1038/s41398-026-04105-3)

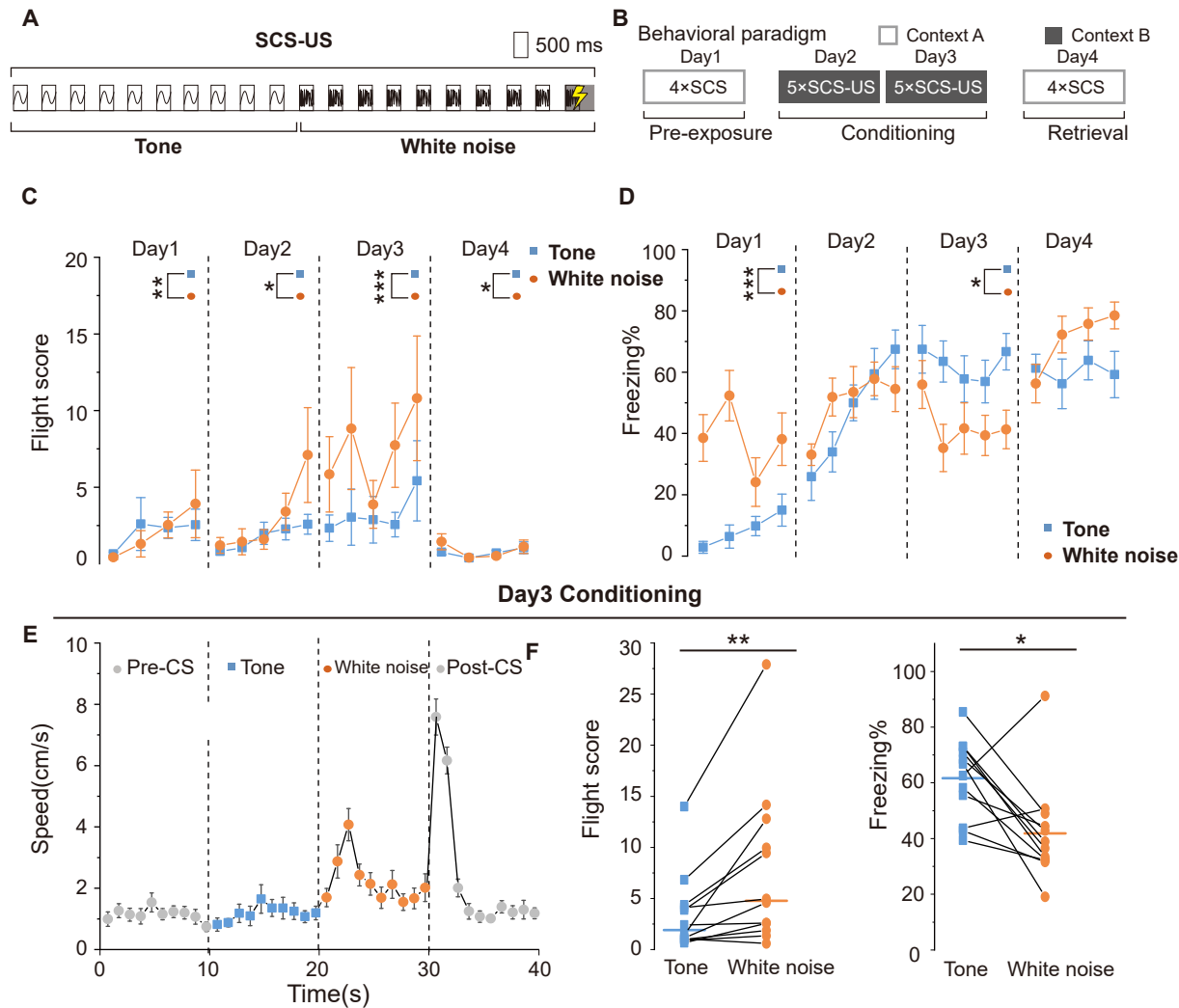

Supplement: Supplementary file 4 — Supplementary Figure 1 [file 41398_2026_4105_MOESM4_ESM.pdf]

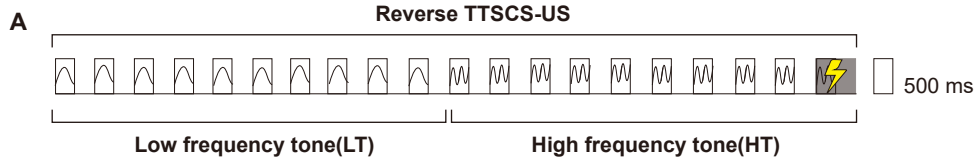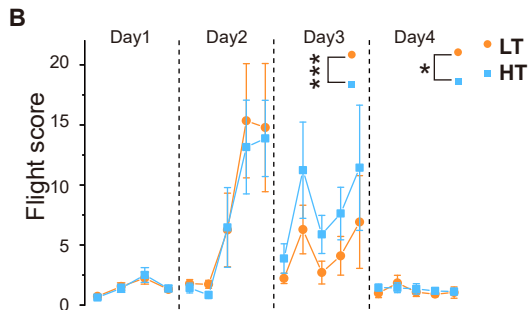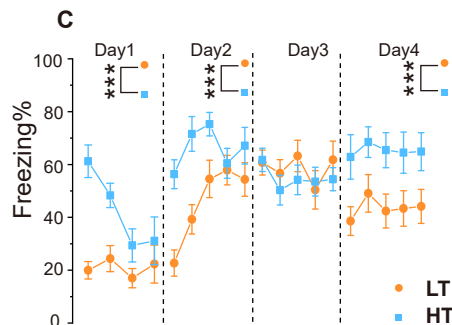

**Day3 Conditioning**

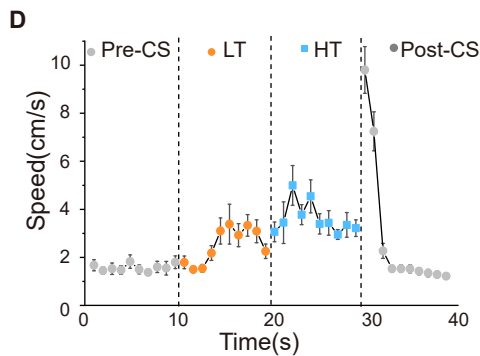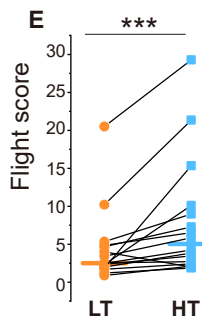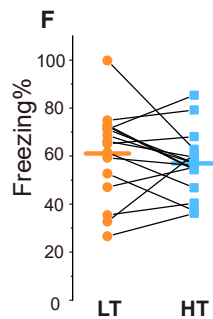

Supplement: Supplementary file 5 — Supplementary Figure 2 [file 41398_2026_4105_MOESM5_ESM.pdf]

# A

## AAV-CaMKII-GCaMP 6s

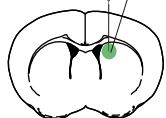

C57/BL6 mouse

# B

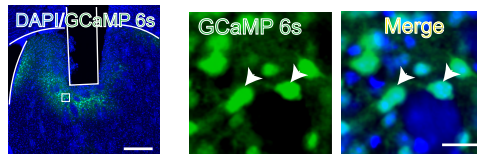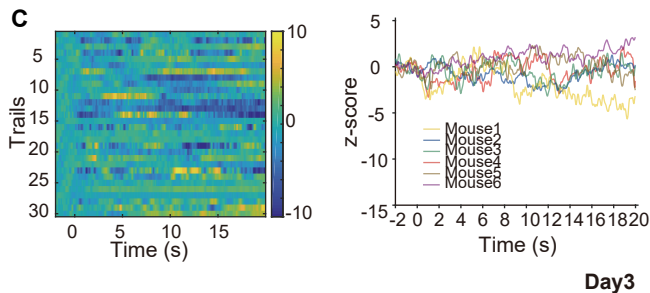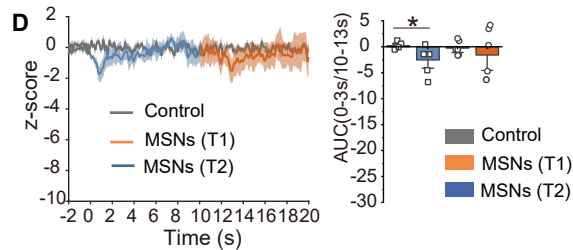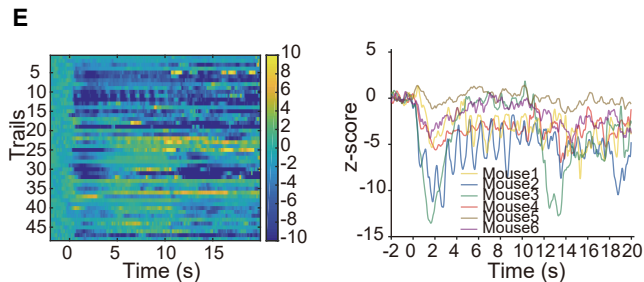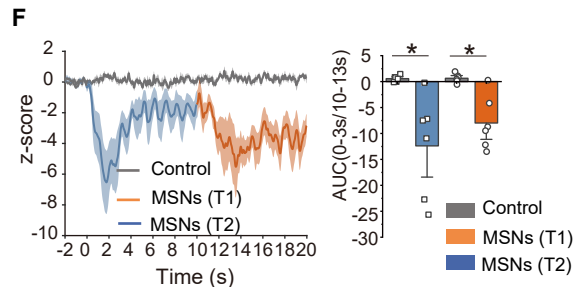

Supplement: Supplementary file 6 — Supplementary Figure 3 [file 41398_2026_4105_MOESM6_ESM.pdf]

T1

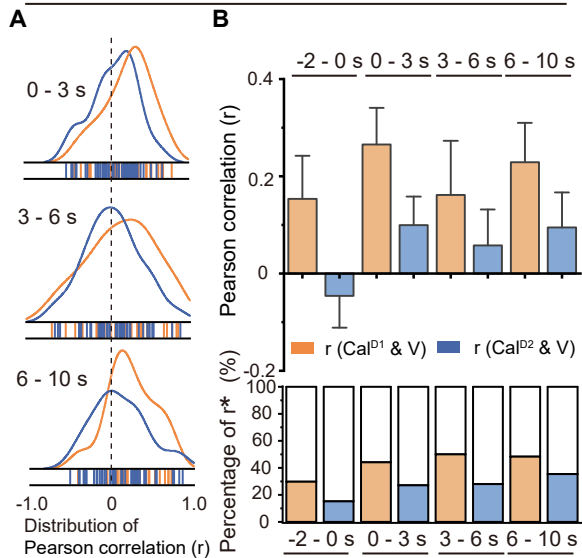

T2

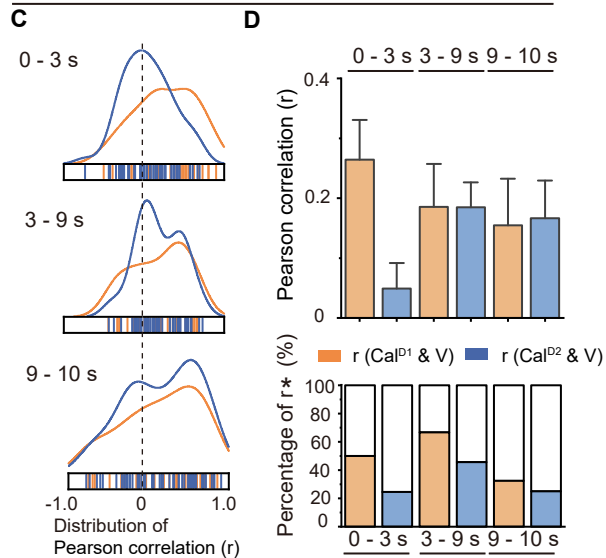

Supplement: Supplementary file 7 — Supplementary Figure 4 [file 41398_2026_4105_MOESM7_ESM.pdf]

**A**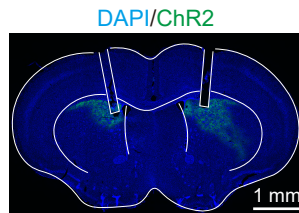

A2a Cre mouse

**B**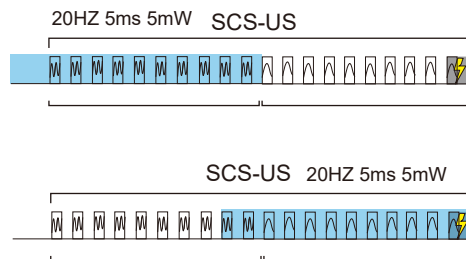**C**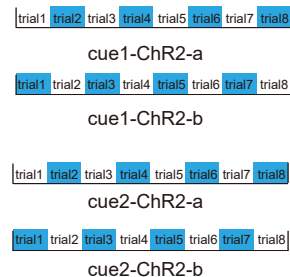**D**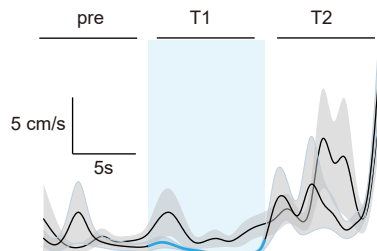**E**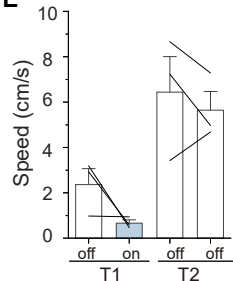**F**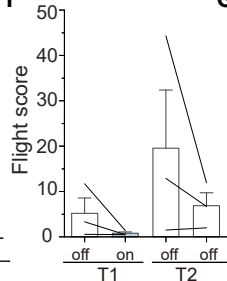**G**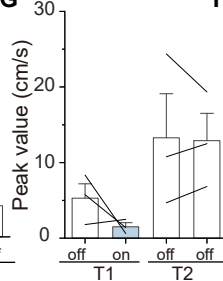**H**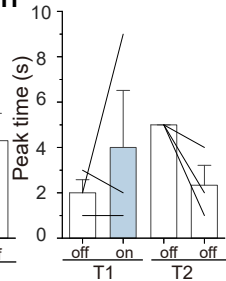**I**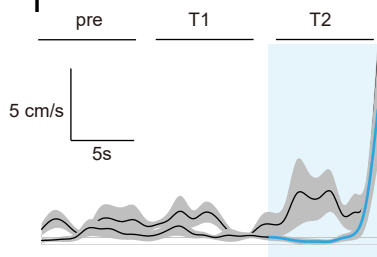**J**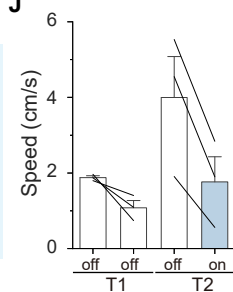**K**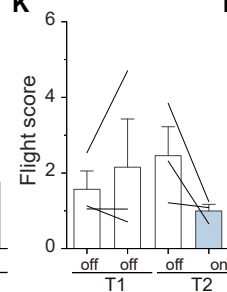**L**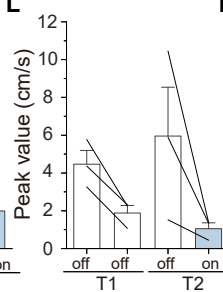**M**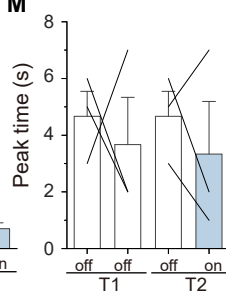

Supplement: Supplementary file 8 — Supplementary Figure 5 [file 41398_2026_4105_MOESM8_ESM.pdf]
